# Transcriptome Profiling Identifies Candidate Genes Associated with the Accumulation of Distinct Sulfur γ-Glutamyl Dipeptides in *Phaseolus vulgaris* and *Vigna mungo* Seeds

**DOI:** 10.3389/fpls.2013.00060

**Published:** 2013-03-25

**Authors:** Dengqun Liao, Dustin Cram, Andrew G. Sharpe, Frédéric Marsolais

**Affiliations:** ^1^Genomics and Biotechnology, Southern Crop Protection and Food Research Centre, Agriculture and Agri-Food CanadaLondon, ON, Canada; ^2^National Research Council CanadaSaskatoon, SK, Canada; ^3^Department of Biology, University of Western OntarioLondon, ON, Canada

**Keywords:** sulfur metabolism, gamma-glutamyl dipeptides, *S*-methylcysteine, *Vigna mungo*, *Phaseolus vulgaris*, developing seed, 454 transcriptome sequencing

## Abstract

Common bean (*Phaseolus vulgaris*) and black gram (*Vigna mungo*) accumulate γ-Glutamyl-*S*-methylcysteine and γ-Glutamyl-methionine in seed, respectively. Transcripts were profiled by 454 pyrosequencing data at a similar developmental stage coinciding with the beginning of the accumulation of these metabolites. Expressed sequence tags were assembled into Unigenes, which were assigned to specific genes in the early release chromosomal assembly of the *P. vulgaris* genome. Genes involved in multiple sulfur metabolic processes were expressed in both species. Expression of *Sultr3* members was predominant in *P. vulgaris*, whereas expression of *Sultr5* members predominated in *V. mungo*. Expression of the cytosolic SERAT1;1 and -1;2 was approximately fourfold higher in *P. vulgaris* while expression of the plastidic SERAT2;1 was twofold higher in *V. mungo*. Among *BSAS* family members, *BSAS4;1*, encoding a cytosolic cysteine desulfhydrase, and *BSAS1;1*, encoding a cytosolic *O*-acetylserine sulphydrylase were most highly expressed in both species. This was followed by *BSAS3;1* encoding a plastidic β-cyanoalanine synthase which was more highly expressed by 10-fold in *P. vulgaris*. The data identify BSAS3;1 as a candidate enzyme for the biosynthesis of *S*-methylcysteine through the use of methanethiol as substrate instead of cyanide. Expression of *GLC1* would provide a complete sequence leading to the biosynthesis of γ-Glutamyl-*S*-methylcysteine in plastids. The detection of *S*-methylhomoglutathione in *P. vulgaris* suggested that homoglutathione synthetase may accept, to some extent, γ-Glutamyl-*S*-methylcysteine as substrate, which might lead to the formation of *S*-methylated phytochelatins. In conclusion, 454 sequencing was effective at revealing differences in the expression of sulfur metabolic genes, providing information on candidate genes for the biosynthesis of distinct sulfur amino acid γ-Glutamyl dipeptides between *P. vulgaris* and *V. mungo*.

## Introduction

Among grain legumes, common bean (dry bean, *Phaseolus vulgaris* L.) is dominant for direct human consumption. Like other grain legumes, its protein quality is limited by the sub-optimal levels of the essential sulfur amino acids, Met, and Cys. Common bean accumulates high levels of a non-protein sulfur amino acid in seed, *S*-methyl-Cys, partly in the form of a γ-Glu dipeptide (Giada et al., 1998; Taylor et al., 2008). γ-Glu-*S*-methyl-Cys accumulates exclusively in seed and not in other tissues (Watanabe et al., 1971). The non-protein amino acid, *S*-methyl-Cys, cannot substitute for Met or Cys in the diet (Padovese et al., 2001). The taxonomic distribution of *S*-methyl-Cys encompasses all *Phaseolus* and several *Vigna* species, notably cowpea (*Vigna unguiculata*), and mung bean (*Vigna radiata*), but excludes black gram (*Vigna mungo*) and adzuki bean (*Vigna angularis*), two Asian *Vigna* species (Baldi and Salamini, 1973; Evans and Boulter, 1975; Kasai and Larsen, 1980; Kasai et al., 1986). *V. mungo* accumulates the dipeptide γ-Glu-Met rather than γ-Glu-*S*-methyl-Cys (Otoul et al., 1975). While *S*-methyl-Cys may be formed by direct methylation of Cys in radish, this does not appear to be the case in common bean (Thompson and Gering, 1966). Rather, it was proposed to be formed by the condensation of *O*-acetylserine and methanethiol, catalyzed by *O*-acetylserine sulfhydrylase (also designated as Cys synthase), a member of the β-substituted Ala synthase (BSAS) family of enzymes. *O*-Acetylserine sulfhydrylase partially purified from common bean seedlings catalyzes this reaction *in vitro* (Smith and Thompson, 1971). This reaction also takes place in *Arabidopsis* cell cultures grown in the presence of a high concentration of Met (Rébeillé et al., 2006). The detection of small amounts of *S*-methylhomoglutathione in *V. radiata* seeds suggests that homoglutathione synthetase may accept to some extent γ-Glu-*S*-methyl-Cys as substrate (Kasai et al., 1986).

In common bean, a deficiency of the major seed storage proteins, the 7S globulin phaseolin, and the lectin phytohemagglutinin results in a shift of sulfur from *S*-methyl-Cys to the Cys and Met pools in protein (Taylor et al., 2008). Lack of the major storage proteins is compensated by increased levels of several sulfur-rich proteins, including the 11S globulin legumin, albumin-2, defensin D1, albumin-1, and Bowman–Birk type proteinase inhibitor (Marsolais et al., 2010; Yin et al., 2011). Analysis of microarray data revealed increased levels of transcripts for two additional types of sulfur-rich proteins, the basic 7S globulin and Kunitz trypsin inhibitor, and a general up-regulation of transcripts coding for sulfate transporters and enzymes of sulfur amino acid metabolism (Liao et al., 2012). Up-regulation of *SERAT1;1* and *-1;2* expression indicated an activation of cytosolic *O*-acetylserine biosynthesis, while down-regulation of *SERAT2;1* encoding a predicted plastidic enzyme suggested that Cys and *S*-methyl-Cys biosynthesis may take place in different subcellular compartments, providing an explanation for their opposite regulation.

In the present study, Roche 454 Genome Sequencer FLX pyrosequencing was utilized to profile the developing seed transcriptome of the two closely related species, *P. vulgaris* and *V. mungo*, both members of subtribe Phaseolinae, which differ by the accumulation of γ-Glu-*S*-methyl-Cys and γ-Glu-Met, respectively. In *P. vulgaris*, this technology has been used to investigate transcripts from leaf, flower, root, and pod (Kalavacharla et al., 2011; Liu et al., 2012), whereas Illumina’s sequencing by synthesis technology has been used to profile the seed transcriptome (Young et al., 2011; Zhai et al., 2011) and identify microRNAs (Peláez et al., 2012). Transcript profiling data generated by high-throughput sequencing has not been reported yet from *V. mungo* but is available from *V. radiata* and *V. unguiculata* (Barrera-Figueroa et al., 2011; Kido et al., 2011; Moe et al., 2011). Differences in the expression of sulfate transporters and enzymes of sulfur amino acid metabolism suggest adaptations which may be related to the accumulation of distinct sulfur amino acid γ-Glu dipeptides in developing seed.

## Materials and Methods

### Plant materials and growth conditions

The common bean (*P. vulgaris*) line BAT93 (Nodari et al., 1993) and black gram (*V. mungo*) cultivar Barimash-2 (Afzal et al., 2002) were grown in growth cabinets (Conviron E8H, Controlled Environments, Winnipeg, MB, Canada). Seeds were sown in small pots (8 cm × 12 cm) containing Pro-Mix PGX (Premier Horticulture, Rivière-du-Loup, QC, Canada). Seedlings were transplanted to larger pots (17 cm × 20 cm) containing Pro-Mix BX (Premier Horticulture). Plants were given 16 h light (300–400 μmol photons m^−2^ s^−1^) and 8 h dark, with a temperature cycling between 18°C and 24°C (Pandurangan et al., 2012). Plants were fertilized with 20:20:20 (Plant-Prod, Laval, QC, Canada) with the equivalent of 0.1 g per pot for Barimash-2 and 0.3 g per pot for BAT93 after transplantation and subsequently every 2 weeks. Developing seeds were harvested, immediately frozen in liquid nitrogen, and stored at -80°C.

### Extraction and quantification of free amino acids

Amino acids were extracted from seeds and quantified by HPLC after derivatization with phenylisothiocyanate as previously described (Yin et al., 2011). For *V. mungo*, the solvent used for extraction, ethanol:water (70:30) was supplemented with 2% (w/v) polyvinylpolypyrrolidone to neutralize phenolic compounds. γ-Glu-Leu, γ-Glu-Met, and *S*-methylhomoglutathione standards were from Bachem Americas (Torrance, CA, USA). Free amino acid profiles were analyzed with CLUSTER 3.0 (de Hoon et al., 2004).

### RNA extraction, cDNA library preparation, and sequencing

RNA was extracted from developing seeds according to Wang and Vodkin (1994). RNA was quantified by spectrophotometry with a NanoDrop 1000 (Thermo Scientific, Wilmington, DE, USA) and its quality evaluated from A_260/280_ ratio and by agarose gel electrophoresis. Total RNA was treated with amplification-grade DNase I (Life Technologies, Burlington, ON, Canada) and extracted with acidic phenol:chloroform. For purification with the Poly(A) Purist MAG Kit, 480 μg of DNaseI treated total RNA was used, and this was followed by treatment with the RiboMinus Plant Kit for RNA-Seq (Life Technologies). For cDNA Rapid Library Prep, 400 ng of the purified samples was used. Three copies per bead were used for emulsion-based clonal amplification and 2 × 10^6^ beads were loaded per half-plate for 454 pyrosequencing on a Genome Sequencer-FLX Instrument using Titanium chemistry (Roche Applied Science, Laval, QC, Canada).

### Sequence analysis

The 454 read pre-processing steps including filtering based on read quality score, adapter trimming, repeat filtering, low-complexity masking, and poly(A/T) removal were done with custom, in-house scripts of the Bioinformatics lab at National Research Council Canada in Saskatoon, SK, Canada. Assembly of the cleaned 454 ESTs was performed with iAssembler v1.3.0 using default parameters: a maximum of 30bp long end clips, a minimum of 40bp overlap, and 97% identity (Zheng et al., 2011). Stand-alone BLAST (2.2.27+) from NCBI was used with a threshold *e*-value < 1 × 10^−10^ (Park et al., 2012). Gene annotation was performed by BLASTX against *Arabidopsis thaliana* TAIR10 proteins (TAIR10_pep_20101214) (Lamesch et al., 2012). The TAIR ID with the top hit was extracted and used for functional annotation and gene ontology classification (Berardini et al., 2004). ESTs were searched by BLASTX against *Glycine max* proteins from the Glyma1 chromosome-based assembly (Gmax_109_peptide.fa)^1^ (Schmutz et al., 2010). Unigenes were associated with specific genes in the *P. vulgaris* genome by BLASTX and BLASTN against the amino acid and gene coding sequences from the early release genome of the common bean (Pvulgaris_v1.0_218_cds.fa) (Goodstein et al., 2012). These sequence data were produced by the United States Department of Energy Joint Genome Institute^2^ in collaboration with United States Department of Agriculture-National Institute of Food and Agriculture. *P. vulgaris* Unigenes were assigned to a gene in the *P. vulgaris* genome on the basis of the top BLASTN hit and sequences were at least 97% identical. *V. mungo* Unigenes were assigned to a *P. vulgaris* gene in the same manner and sequences were at least 88% identical. BLASTX results were used to confirm the assignments. When multiple Unigenes were assigned to a single gene, the number of reads associated with each of the Unigenes was summed to provide the total number of reads for this gene. When multiple Unigenes were assigned to a single gene, their alignment was usually non-overlapping, i.e., there were gaps in coverage of the reference coding sequence. A nomenclature was developed for sulfate transporters and sulfur metabolic genes on the basis of phylogenetic clustering with *Arabidopsis* and soybean homologs (Yi et al., 2010; Hell and Wirtz, 2011; Takahashi et al., 2011; Liao et al., 2012).

### Accession numbers

Sequencing data are available at SRA^3^ under the accession numbers SRS360251 for *P. vulgaris* and SRS360666 for *V. mungo*. The *P. vulgaris* data is composed of two subsets: GIWE0SV (accession number SRR553466) and GMAPAYS (accession number SRR553467). Only the GMAPAYS subset was used for assembly and data analysis as the GIWE0SV dataset was the result of an additional trial run.

## Results

### Metabolic profiling of free amino acids during seed development in *V. mungo*

**Figure 1:**
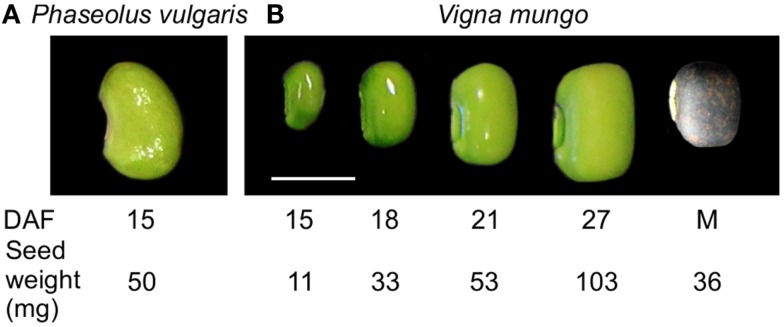
**Seed developmental stages used for transcript and free amino acid profiling**. **(A)** *P. vulgaris* BAT93, stage IV (cotyledon), 15 days after fertilization (DAF), 50 mg seed weight. **(B)** *V. mungo* Barimash-2, 15 DAF (11 mg seed weight), 18 DAF (33 mg), 21 DAF (52 mg), 27 DAF (103 mg), and mature seed (36 mg). Scale bar is equal to 0.5 cm.

**Figure 2:**
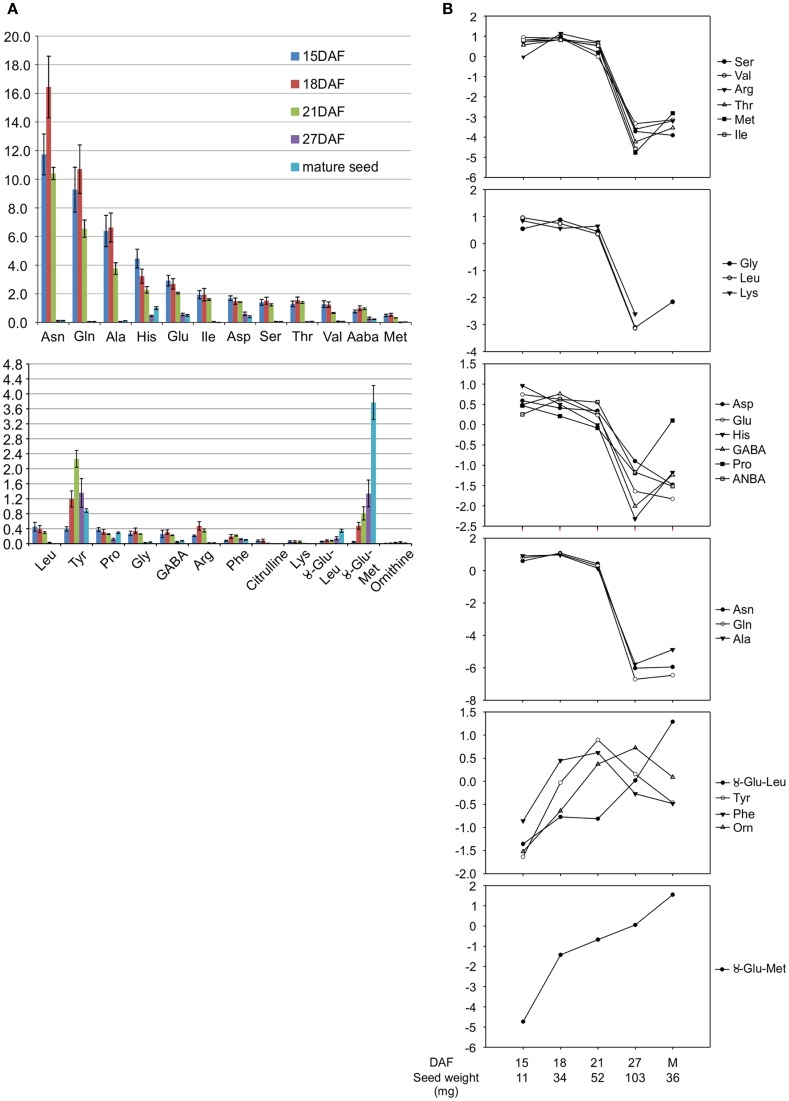
**Free amino acid profiles during seed development in *V. mungo***. **(A)** Free amino acid concentration expressed in nmol per mg seed weight; *n* = 3. Error bars represent standard deviation **(B)** *k*-Means cluster analysis of free amino acid profiles during seed development. Data on the *y* axis is the amino acid concentration in **(A)** normalized to the average of developmental stages and expressed in a log_2_ scale.

Stage IV, corresponding to the cotyledon stage, with an average seed weight of 50 mg, at 15 days after flowering (DAF) (Figure 1) (Walbot et al., 1972) was selected for transcript profiling of developing *P. vulgaris* seeds because it corresponds to the beginning of the active phase of γ-Glu-*S*-methyl-Cys accumulation, before free *S*-methyl-Cys levels start to decline (Yin et al., 2011). To identify a physiologically similar stage in *V. mungo*, free amino acids, including the dipeptides γ-Glu-Leu and γ-Glu-Met (Otoul et al., 1975), were extracted and profiled at five stages of seed development (Figure 1). Figure 2A shows that Asn, followed by Gln, and Ala, were the dominant free amino acids at early stages of development, while γ-Glu-Met was the most abundant at maturity. Amino acid concentration was normalized over the average of developmental stages, expressed in a log_2_ scale, and *k*-means analysis performed to reveal common developmental patterns. Amino acids were grouped into six clusters (Figure 2B). The levels of most amino acids, present in the first four clusters, were stable from 15 to 21 DAF and declined abruptly at 27 DAF suggesting their incorporation into storage proteins. The fifth cluster grouped amino acids with levels similar or greater than average at 27 DAF and included Phe, Tyr, Orn, as well as γ-Glu-Leu, whose levels increased steadily except between 18 and 21 DAF. γ-Glu-Met was clustered separately from other amino acids because its levels increased at all developmental stages. Eighteen DAF was selected for transcript profiling as it corresponded to the beginning of the active phase of γ-Glu-Met accumulation when free Met was at its highest levels. In *P. vulgaris*, storage protein transcripts are most abundant beginning at stage IV, which precedes storage product accumulation (Bobb et al., 1995), whereas phases of cell expansion and synthesis of reserve metabolites more completely overlap in *V. mungo* (Sehgal and Gandhi, 1987).

### Quantification of *S*-methylhomoglutathione in *P. vulgaris* seeds

To determine whether *S*-methylhomoglutathione accumulates in *P. vulgaris* as in *V. radiata* (Kasai et al., 1986), free amino acids were extracted from mature seeds of the BAT93 line. *S*-Methylhomoglutathione was quantified in comparison with a synthetic standard. The concentration measured was equal to 73.3 ± 6.8 pmol per mg seed weight (average ± standard deviation; *n* = 3), and is similar to that determined in *V. radiata* seeds (Kasai et al., 1986).

### Transcript profiling of developing seeds from *P. vulgaris* and *V. mungo* by 454 pyrosequencing

**Figure 3:**
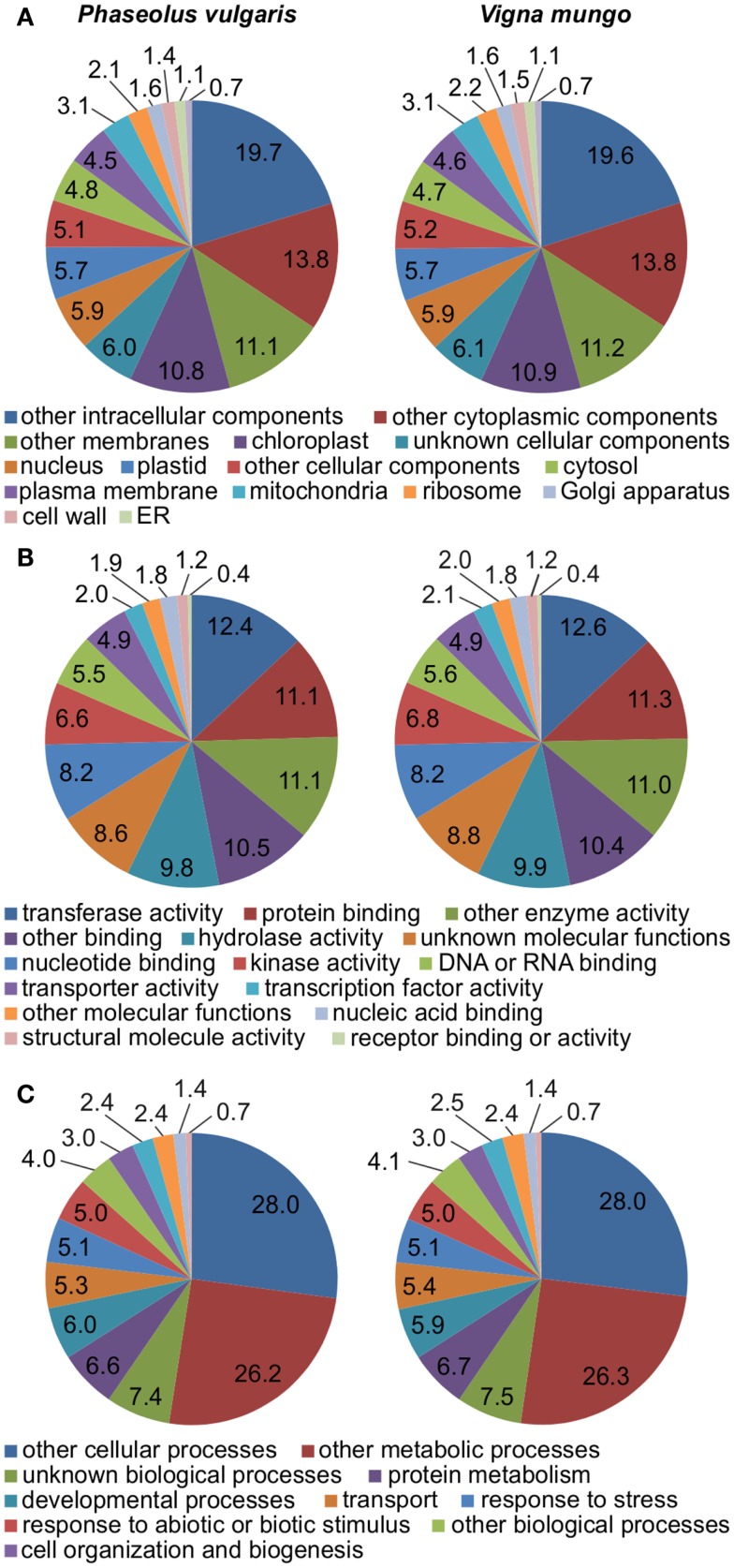
**Representation of gene ontology categories in the transcriptomes of *P. vulgaris* and *V. mungo***. The percentage of Unigenes present in a gene ontology category is indicated for **(A)** cellular component; **(B)** molecular function; and **(C)** biological process.

RNA was extracted from developing seeds of *P. vulgaris* and *V. mungo* at the selected developmental stages. Transcripts were profiled on a 454 Genome Sequencer-FLX Titanium platform. A half-plate was sequenced for each sample starting from the same amount of input cDNA. Table 1 lists general statistics for the 454 transcriptome data. The number of expressed sequenced tags (ESTs) after pre-processing was remarkably similar between the two species, within a 0.3% difference. *V. mungo* ESTs were slightly longer on average by 9%. The number of assembled contigs was very close between the two species, within a 5% difference. The number of singletons was considerably higher in *P. vulgaris*, by 38%, which correlated with the shorter EST length. Importantly, the number of Unigenes with significant BLAST hits to the *Arabidopsis*, soybean, and common bean genomes was almost equal between the two species. An analysis of gene ontology categories showed a comparable distribution between the two samples (Figure 3). Overall, these data suggested a similar physiological state between samples. These data validated the selection of developmental stages from the two species, and supported further comparative analyzes of the expression of sulfur metabolic genes.

**Table 1:** **Summary of 454 transcriptome data from *P. vulgaris* and *V. mungo* developing seeds**.

	*P. vulgaris*	*V. mungo*
Number of reads	757719	717036
Number of ESTs after pre-processing	662417	660318
Median length (bp)	358	408
Average length (bp)	339	369
Longest length (bp)	770	665
Mean GC content (%)	45.1	44.7
Number of contigs	38344	40125
Number of singletons	39104	28349
Number of Unigenes	77448	68474
Contig average length (bp)	751	809
Unigene average length (bp)	510	583
Longest contig (bp)	7845	6749
Number of Unigenes with BLASTX hit to TAIR10	40129	41430
Number of Unigenes with BLASTX hit to Glyma1	48524	48571
Number of Unigenes with BLASTX hit to *P. vulgaris* v1.0	51119	50165

*Expressed sequence tags (ESTs) were assembled with iAssembler v1.3.0. BLAST hits were considered significant at an *e*-value < 1 × 10^−10^*.

### Expression of sulfate transporter and sulfur metabolic transcripts

**Figure 4:**
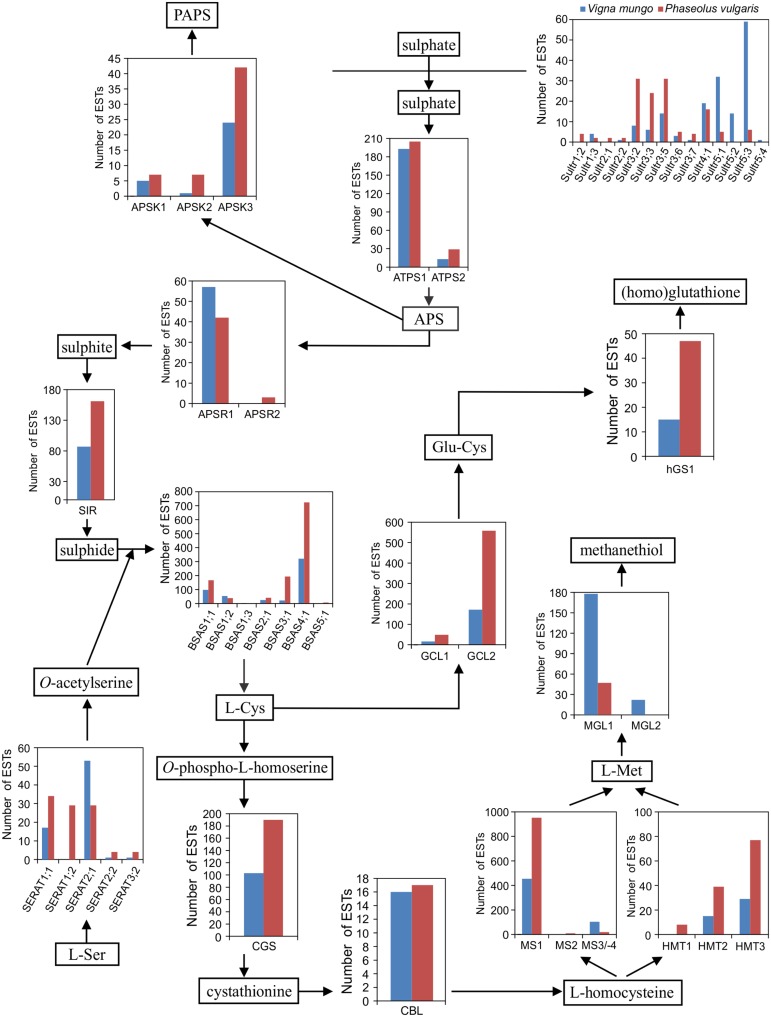
**Expression of sulfate transporter and sulfur metabolic genes in developing seed of *P. vulgaris* and *V. mungo***. ESTs were assigned to individual genes in the chromosomal assembly of the early release of the *P. vulgaris* genome. Abbreviations are as follows: Sultr: sulfate transporter; ATPS: sulfate adenylyltransferase; APS: adenosine 5′-phosphosulfate; APSK: adenylyl-sulfate kinase; PAPS: 3′-phosphoadenosine 5′-phosphosulfate; APSR: adenylyl-sulfate reductase; SIR: sulfite reductase; SERAT: Ser acetyltransferase; BSAS: β-substituted Ala synthase; CGS: cystathionine γ-synthase; CBL: cystathionine β-lyase; HMT: homocysteine *S*-methyltransferase; MS: Met synthase; MGL: Met γ-lyase; GCL: Glu-Cys ligase; hGS: homoglutathione synthetase.

To investigate possible differences in expression of sulfate transporter or sulfur metabolic genes, which may correlate with the accumulation of distinct sulfur amino acid γ-Glu dipeptides, Unigenes, and their corresponding ESTs were assigned to specific genes in the early release chromosomal assembly of the *P. vulgaris* genome. A previously developed nomenclature based on phylogenetic clustering with *Arabidopsis* and soybean homologs was utilized (Table A1 in Appendix) (Liao et al., 2012). Sulfate transporter and sulfur metabolic genes are often members of multigene families and the corresponding gene products may have different subcellular localizations. Table 2 compares the numbers of genes in the *P. vulgaris*, *G. max*, and *Arabidopsis* genomes coding for sulfate transporters and the different classes of sulfur metabolic enzymes. The higher number of genes in *G. max* reflects its paleopolyploid nature as compared with the true diploids *P. vulgaris* and *Arabidopsis*. As compared with *Arabidopsis*, *P. vulgaris* has more genes coding for sulfate transporters and enzymes of glutathione/homoglutathione biosynthesis, but fewer genes involved in sulfate assimilation or activation (Table A1 in Appendix). Figure 4 presents the number of ESTs assigned to each gene in the sulfur metabolic pathway. This figure shows that genes involved in multiple sulfur metabolic processes, including sulfate transport, sulfate activation, sulfate assimilation, *de novo* Cys and Met biosynthesis, homoglutathione biosynthesis and Met catabolism were expressed in both species. Transcripts for 12 different sulfate transporter genes were detected in the *P. vulgaris* and *V. mungo* samples, out of a total of 19 encoded by the *P. vulgaris* genome. Transcripts of members of the *Sultr1* and *-2* families, encoding high and low affinity sulfate transporters involved in sulfate uptake by the root, and in the translocation of sulfate from root to shoot, respectively (Takahashi et al., 2000; Yoshimoto et al., 2007; Barberon et al., 2008), were detected at very low levels. Rather, group 3 genes were the most highly expressed in *P. vulgaris*, with 95 ESTs representing five different genes, as compared with32 ESTs assigned to the same genes in *V. mungo*. Group 3 genes have been associated with the transfer of sulfate from the seed coat to the embryo in *Arabidopsis* (Zuber et al., 2010). Transcripts of group 5 genes were dominant in *V. mungo*, with 106 ESTs for four different genes, as compared with11 ESTs for the same genes in *P. vulgaris*. Sultr5;2 has been implicated as a high affinity molybdate transporter in *Arabidopsis* (Tomatsu et al., 2007; Baxter et al., 2008), although expression of the corresponding gene in wheat under sulfur deficiency did not correlate with molybdenum accumulation (Shinmachi et al., 2010). The present data suggest that at least some of the Sultr5 members may function as sulfate transporters in *V. mungo* seeds.

**Table 2:** **Number of genes coding for sulfate transporters and sulfur metabolic enzymes in the genomes of *P*. vulgaris, *G. max*, and Arabidopsis**.

	*P. vulgaris*	*G. max*	Arabidopsis
Sulfate transporter	19	35	14
Sulfate adenylyl- transferase	2	4	4
Adenylyl-sulfate kinase	3	5	4
Adenylyl-sulfate reductase	2	3	3
Sulfite reductase	1	2	1
Sulfite oxidase	1	2	1
Serine acetyltransferase	6	9	5
β-Substituted Alasynthase	8	18	9
Cystathionine γ-synthase	1	2	2
Cystathionine β-lyase	1	2	1
Homocysteine *S*-methyltransferase	3	4	3
Metsynthase	3	7	3
Met γ-lyase	2	3	1
Glu-Cys ligase	2	4	1
Glutathione synthetase	3	3	1

The first step in sulfate assimilation is catalyzed by sulfate adenylyltransferase (ATPS). Its product, adenosine 5′-phosphosulphate, is found at a branch point between sulfate activation and reductive sulfate assimilation (Mugford et al., 2009, 2011; Kopriva et al., 2012). Among the two *ATPS* genes, *ATPS1* was predominantly expressed in both species. Adenylyl-sulfate kinase (APSK) is responsible for the biosynthesis of the universal sulfate donor, 3′-phosphoadenosine 5′-phosphosulphate. Of the three *APSK* genes, *APSK3* was the most highly expressed. In the other branch of the pathway leading to reductive sulfate assimilation, adenylyl-sulfate reductase (APSR) produces sulfite from adenosine 5′-phosphosulphate. *APSR1*was the main isoform expressed in seed. *APSR2* was expressed at lower levels, by more than 10-fold and detected only in *P. vulgaris*. Sulfite reductase (SIR) is encoded by a single gene in *P. vulgaris* and its expression was detected in both species. The same was observed for sulfite oxidase, with 14 ESTs in *P. vulgaris* as compared with 27 in *V. mungo*. Overall, the genes involved in sulfate assimilation and activation were expressed at relatively similar levels between *P. vulgaris* and *V. mungo*.

Sulfide is combined with *O*-acetylserine produced by serine acetyltransferase (SERAT) to form Cys, in a reaction catalyzed by *O*-acetylserine sulfhydrylase. *P. vulgaris* had a higher number of ESTs for SERAT1;1 and -1;2 than *V. mungo* by close to fourfold, while transcripts for SERAT2;1 were more abundant in *V. mungo* by approximately twofold. According to phylogeny and analysis with WoLF PSORT (Horton et al., 2007), SERAT1;1 and -1;2 are predicted to be localized in the cytosol and SERAT2;1 in plastids. *Arabidopsis* has two SERAT2 isoforms localized in plastids and mitochondria, respectively (Noji et al., 1998), and a fusion between soybean SERAT2;1 and a fluorescent protein was detected in plastids and the cytosol (Liu et al., 2006). Among BSAS family members, *BSAS4;1* was the most abundantly expressed followed by *BSAS1*;*1*. The number of ESTs associated with these two genes was approximately twofold higher in *P. vulgaris* than in *V. mungo*. *BSAS1;1* encodes a cytosolic *O*-acetylserine sulphydrylase in both soybean and *Arabidopsis* (Heeg et al., 2008; Watanabe et al., 2008; Hell and Wirtz, 2011; Yi and Jez, 2012; Yi et al., 2012). *BSAS4;1* clusters with *GmBSAS4;2*, encoding a cytosolic BSAS which preferentially catalyzes the desulfhydration of Cys (GmDES; Glyma10g30140) (Yi et al., 2010; Yi and Jez, 2012). *Arabidopsis* BSAS4;3 also represents a Cys desulfhydrase (Alvarez et al., 2010). *BSAS3;1* was also abundantly expressed in *P. vulgaris*, with a number of ESTs approximately ninefold higher than in *V. mungo*. In Arabidopsis, BSAS3;1 is a β-cyanoalanine synthase involved in the detoxication of cyanide. This mitochondrial enzyme catalyzes the formation of β-cyanoalanine from Cys and cyanide releasing sulfide (Watanabe et al., 2008). Likewise, soybean BSAS3;1 is a β-cyanoalanine synthase (Yi and Jez, 2012; Yi et al., 2012). Interestingly, common bean BSAS3;1 is predicted by WoLF PSORT to be localized in plastids rather than mitochondria.

In the steps leading to the formation of Met, Cys is condensed by cystathionine γ-synthase (CGS) with *O*-phospho-l-homoserine to form cystathionine. Cystathionine is then cleaved by cystathionine β-lyase (CBL) producing homocysteine which can be utilized for Met biosynthesis. Both steps catalyzed by CGS and CBL occur in plastids. Homocysteine can be methylated by homocysteine *S*-methyltransferase using *S*-methyl-Met as methyl donor, a major form of organic sulfur transported to seed (Bourgis et al., 1999; Ranocha et al., 2000; Lee et al., 2008; Tan et al., 2010). Alternatively, homocysteine can be methylated by Met synthase (MS) using methyltetrahydrofolate as methyl donor. MS is present in both plastids and the cytosol. However, it is assumed that only plastidic MS has a significant contribution to *de novo* Met biosynthesis, while cytosolic MS is involved in the recycling of Met as part of the *S*-adenosyl-Met cycle (Amir and Hacham, 2008; Jander and Joshi, 2010). Both CGS and CBL are encoded by unique genes in *P. vulgaris*. The number of ESTs was almost twofold higher for *CGS* in *P. vulgaris* than in *V. mungo*, but similar for *CBL*. Among the three *HMT* genes, *HMT3* was predominantly expressed in both species, followed by *HMT2* and *-1*. The number of ESTs was higher in *P. vulgaris* for all three genes, on average by almost threefold. Among *MS* genes, *MS1*, encoding a predicted cytosolic enzyme, was the most highly expressed, with a twofold higher number of ESTs in *P. vulgaris* than in *V. mungo*. ESTs corresponding to the plastidic MS were more abundant in *V. mungo*, by approximately fivefold. In the early release chromosomal assembly of the *P. vulgaris* genome, the gene coding for plastidic MS is split into two, *MS3*, encoding an N-terminal segment and *MS4*, encoding a C-terminal segment, located on chromosomes 5 and 6, respectively. The present EST data do not support this split since a contig overlapped between the two segments, which suggests a possible artifact from the genome assembly process. The enzyme Met γ-lyase catalyzes the breakdown of Met, producing methanethiol, ammonia, and 2-oxobutanoate, which can be utilized for Ile biosynthesis (Rébeillé et al., 2006; Goyer et al., 2007; Joshi and Jander, 2009). There are two *MGL* genes in the *P. vulgaris* genome. Only *MGL1* was expressed in *P. vulgaris*, while both genes were expressed in *V. mungo*. The number of ESTs for *MGL1* was approximately threefold higher in *V. mungo*.

Beside Met biosynthesis, Cys can be used to synthesize glutathione and homoglutathione (Galant et al., 2011). The enzyme γ-Glu-Cys ligase (GCL) catalyzes the formation of γ-Glu-Cys. There are two *GCL* genes in the *P. vulgaris* genome. GCL1 is predicted to be localized in the chloroplast and GCL2 in the cytosol, in agreement with subcellular localization data from spinach and pea leaf, and common bean and cowpea nodule (Hell and Bergmann, 1990; Moran et al., 2000). *GLC2* was expressed at higher levels than *GCL1* in both species, by approximately 10-fold. Both *GCL1* and *-2* were expressed at higher levels in *P. vulgaris* than in *V. mungo*, by approximately threefold. The *P. vulgaris* genome contains a single gene encoding homoglutathione synthetase (*hGS1*) and two genes encoding glutathione synthetase (*GS1* and *-2*) located in tandem at a unique locus, like in other legumes (Frendo et al., 2001). Only *hGS1* transcripts were detected in seed, and not those of *GS1* and *-2*. This result is consistent with the fact that the concentration of homoglutathione is 80-fold higher than the concentration of glutathione in *P. vulgaris* seeds (Klapheck, 1988). The number of *hGS1* ESTs was approximately threefold higher in *P. vulgaris* than in *V. mungo*. hGS1 protein is predicted to be localized in the cytosol, consistent with previous reports on the subcellular localization of hGS activity in *Phaseolus coccineus* leaves and nodules of common bean (Klapheck et al., 1988; Moran et al., 2000). Phytochelatin synthase (PCS) uses homoglutathione to synthesize phytochelatins. There are two PCS genes in the *P. vulgaris* genome located in tandem. Both were expressed at low levels, with 1 EST each for *PCS1* in both species, while four *PCS2* ESTs were detected exclusively in *P. vulgaris*.

## Discussion

The transcriptome profiling data reflects the complexity of sulfur metabolism at the cellular level. Despite the close relationship between species, and the similarity in physiological, biochemical, and gene expression profiles between samples, key differences were noted in the number of ESTs assigned to different sulfur metabolic genes. These differences in gene expression may reflect adaptations related to the accumulation of distinct sulfur γ-Glu dipeptides. The data may provide information on potential candidate genes involved in their biosynthesis.

A prior study focusing on gene expression analysis between a pair of related *P. vulgaris* genotypes contrasted in the accumulation of Cys and *S*-methyl-Cys indicated that increased levels of Cys were associated with enhanced gene expression of cytosolic SERAT1;1 and -1;2, while lower levels of *S*-methyl-Cys were associated with decreased transcript levels for plastidic SERAT2;1 (Liao et al., 2012). This finding suggested a spatial separation of Cys and *S*-methyl-Cys biosynthesis in different subcellular compartments, the cytosol, and plastids, respectively. These data appeared consistent with the fact that legume seeds rely primarily on cytosolic *de novo* Cys biosynthesis. Indeed, overexpression of a cytosolic *O*-acetylserine sulfhydrylase in soybean seed led to increased Cys levels (Kim et al., 2012), whereas overexpression of a plastidic SERAT did not alter sulfur amino acid concentration in chickpea seed (Tabe et al., 2010). In agreement with these findings, *BSAS1;1*, encoding a cytosolic *O*-acetylserine sulfhydrylase, was among the most highly expressed members of the BSAS family. Surprisingly, the member with the highest expression, *BSAS4;1* encodes a cytosolic Cys desulfhydrase. The significance of its high expression in seed is not clear. The largest difference in expression between species was observed for *BSAS3;1* encoding a β-cyanoalanine synthase predicted to be localized in plastids. Interestingly, β-cyanoalanine synthase purified from aerial tissues of *Lathyrus latifolius* and from leaves of *Spinacia oleracea* catalyzed the formation of *S*-methyl-Cys from Cys and methanethiol at a relatively low rate corresponding to 1–4% of β-cyanoalanine synthase activity (Ikegami et al., 1988a,b). This activity reached 13% for the β-cyanoalanine synthase purified from developing seeds of *Vicia angustifolia* (Ikegami et al., 1989). Taken together, the previous implication of plastidial SERAT2;1, the approximately 10-fold higher expression of *BSAS3;1* in *P. vulgaris* as compared with *V. mungo*, encoding a plastidic β-cyanoalanine synthase, and the fact that this enzyme may catalyze a similar reaction with methanethiol as with hydrogen cyanide, leading to the formation of *S*-methyl-Cys suggests that BSAS3;1 may be responsible for the formation of *S*-methyl-Cys in *P. vulgaris* seed. Met γ-lyase may provide a source of methanethiol for *S*-methyl-Cys biosynthesis. Contrary to expectations, a higher number of ESTs for *MGL* genes were observed in *V. mungo* than in *P. vulgaris*.

The enzyme γ-GCL may be involved in the biosynthesis of γ-Glu-*S*-methyl-Cys. The mammalian enzyme has substantial *in vitro* activity with *S*-methyl-Cys as substrate (Rathbun, 1967; Sekura and Meister, 1977). The presence of a plastidic γ-GCL encoded by *GCL1* expressed in *P. vulgaris* seed would enable a complete pathway of γ-Glu-*S*-methyl-Cys biosynthesis in this organelle. The presence of a low concentration of *S*-methylhomoglutathione suggests that hGS1 may accept, at least to a limited extent, γ-Glu-*S*-methyl-Cys as substrate. *In vitro* data indicates that PCS1 can utilize *S*-alkylated glutathione for the synthesis of *S*-alkyl phytochelatins with a high efficiency (Vatamaniuk et al., 2000). This suggests that some *S*-methyl-Cys may accumulate in *S*-methylated phytochelatins, which may account, at least in part, for the discrepancy between concentrations measured for γ-Glu-*S*-methyl-Cys and total *S*-methyl-Cys in *P. vulgaris* seed (Taylor et al., 2008).

In summary, 454 transcriptome sequencing has been used to investigate the expression of sulfur metabolic genes in developing seeds of related *Phaseolina*e species accumulating a different sulfur γ-Glu dipeptide. Despite an overall similar physiological, biochemical, and gene expression profile between the two species, this technique was efficient at revealing differences in the expression of sulfur metabolic genes which provide information on candidate genes for the biosynthesis of these metabolites.

## Conflict of Interest Statement

The authors declare that the research was conducted in the absence of any commercial or financial relationships that could be construed as a potential conflict of interest.

## Footnotes

^1^http://www.phytozome.net/commonbean_er

^2^http://www.jgi.doe.gov/

^3^http://www.ncbi.nlm.nih.gov/sra/

## Appendix

**Table A1:** **Sulfate transporter and sulfur metabolic genes in the *P. vulgaris* genome**.

Gene name	Accession
*Sultr1;1*	Phvul.006G207800
*Sultr1;2*	Phvul.009G028500
*Sultr1;3*	Phvul.008G170800
*Sultr2;1*	Phvul.009G028400
*Sultr2;2*	Phvul.008G170700
*Sultr2;3*	Phvul.001G250700
*Sultr2;4*	Phvul.001G250800
*Sultr3;1*	Phvul.001G154200
*Sultr3;2*	Phvul.007G174100
*Sultr3;3*	Phvul.004G161600
*Sultr3;4*	Phvul.002G095200
*Sultr3;5*	Phvul.002G095300
*Sultr3;6*	Phvul.010G151000
*Sultr3;7*	Phvul.005G171800
*Sultr4;1*	Phvul.008G015600
*Sultr5;1*	Phvul.010G152700
*Sultr5;2*	Phvul.009G098800
*Sultr5;3*	Phvul.001G056100
*Sultr5;4*	Phvul.001G056300
*APS1*	Phvul.007G062900
*APS2*	Phvul.004G045400
*APSK1*	Phvul.002G010900
*APSK2*	Phvul.003G235300
*APSK3*	Phvul.001G111000
*APSR1*	Phvul.006G149200
*APSR2*	Phvul.003G079800
*SOX*	Phvul.004G054200
*SIR*	Phvul.011G021800
*SERAT1;1*	Phvul.001G170600
*SERAT1;2*	Phvul.010G110600
*SERAT2;1*	Phvul.006G055200
*SERAT2;2*	Phvul.008G277800
*SERAT3;2*	Phvul.002G114700
*SERAT3;1*	Phvul.003G269000
*BSAS1;1*	Phvul.002G045200
*BSAS1;2*	Phvul.006G099100
*BSAS1;3*	Phvul.007G057600
*BSAS2;1*	Phvul.003G060200
*BSAS3;1*	Phvul.008G061100
*BSAS4;1*	Phvul.007G185200
*BSAS4;2*	Phvul.007G185100
*BSAS5;1*	Phvul.001G107500
*CGS*	Phvul.008G038100
*CBL*	Phvul.001G125400
*HMT1*	Phvul.003G036100
*HMT2*	Phvul.001G153900
*HMT3*	Phvul.007G060300
*MS1*	Phvul.001G113800
*MS2*	Phvul.004G051600
*MS3*	Phvul.005G048200
*MS4*	Phvul.006G007600
*MGL1*	Phvul.001G082000
*MGL2*	Phvul.004G090200
*GCL1*	Phvul.002G289200
*GCL2*	Phvul.002G157600
*hGS1*	Phvul.006G094500
*GS1*	Phvul.006G094600
*GS2*	Phvul.006G094700
*PCS1*	Phvul.001G162600
*PCS2*	Phvul.001G162700
